# Assessment of prenatal cerebral and cardiac metabolic changes in a rabbit model of fetal growth restriction based on ^13^C-labelled substrate infusions and *ex vivo* multinuclear HRMAS

**DOI:** 10.1371/journal.pone.0208784

**Published:** 2018-12-27

**Authors:** Rui V. Simões, Miquel E. Cabañas, Carla Loreiro, Miriam Illa, Fatima Crispi, Eduard Gratacós

**Affiliations:** 1 Fetal i+d Fetal Medicine Reseach Center, BCNatal—Barcelona Center for Maternal-Fetal and Neonatal Medicine (*Hospital Clínic and Hospital Sant Joan de Déu*), ICGON, IDIBAPS, University of Barcelona, Centre for Biomedical Research on Rare Diseases (CIBER-ER), Barcelona, SPAIN; 2 *Servei de Resonància Magnètica Nuclear*, *Universitat Autònoma de Barcelona* (UAB), SPAIN; Loma Linda University School of Medicine, UNITED STATES

## Abstract

**Background:**

We have used a previously reported rabbit model of fetal growth restriction (FGR), reproducing perinatal neurodevelopmental and cardiovascular impairments, to investigate the main relative changes in cerebral and cardiac metabolism of term FGR fetuses during nutrient infusion.

**Methods:**

FGR was induced in 9 pregnant New Zealand rabbits at 25 days of gestation: one horn used as FGR, by partial ligation of uteroplacental vessels, and the contralateral as control (appropriate for gestation age, AGA). At 30 days of gestation, fasted mothers under anesthesia were infused i.v. with 1-^13^C-glucose (4 mothers), 2-^13^C-acetate (3 mothers), or not infused (2 mothers). Fetal brain and heart samples were quickly harvested and frozen down. Brain cortex and heart apex regions from 30 fetuses were studied *ex vivo* by HRMAS at 4°C, acquiring multinuclear 1D and 2D spectra. The data were processed, quantified by peak deconvolution or integration, and normalized to sample weight.

**Results:**

Most of the total ^13^C-labeling reaching the fetal brains/hearts (80–90%) was incorporated to alanine and lactate (cytosol), and to the glutamine-glutamate pool (mitochondria). Acetate-derived lactate (Lac C2C3) had a slower turnover in FGR brains (~ -20%). In FGR hearts, mitochondrial turnover of acetate-derived glutamine (Gln C4) was slower (-23%) and there was a stronger accumulation of phospholipid breakdown products (glycerophosphoethanolamine and glycerophosphocholine, +50%), resembling the profile of non-infused control hearts.

**Conclusions:**

Our results indicate specific functional changes in cerebral and cardiac metabolism of FGR fetuses under nutrient infusion, suggesting glial impairment and restricted mitochondrial metabolism concomitant with slower cell membrane turnover in cardiomyocytes, respectively. These prenatal metabolic changes underlie neurodevelopmental and cardiovascular problems observed in this FGR model and in clinical patients, paving the way for future studies aimed at evaluating metabolic function postnatally and in response to stress and/or treatment.

## Introduction

Fetal growth restriction (FGR) due to placental insufficiency is associated with sustained hypoxemia and undernutrition of the growing fetus, and affects up to 10% of gestations [1, 2]. FGR has been associated with suboptimal neurodevelopment [3], including brain structural and metabolic changes at pre- [4, 5] and post-natal [6–8] ages, and with cardiac remodeling and dysfunction, detected from fetal life [9–12] and predisposing for cardiovascular disease in adult life [13]. Despite the extensive knowledge about the clinical effects of FGR, there is still a poor understanding of its pathophysiological basis. This is more readily addressable in preclinical animal models.

A rabbit model of FGR based on the selective ligation of utero-placental vessels has been previously reported [14]. Although not recreating a placental insufficiency *per se*, this model reproduces several perinatal features of its clinical condition and leads to neurobehavioral impairment [15, 16]. Thus, smaller brain sizes and microstructural changes in white matter have been reported at birth [15], leading to long term changes in brain organization [16, 17]. The model also reproduces closely the cardiovascular Doppler changes described in humans [18], with fetal hearts presenting a more globular shape and permanent sarcomere changes [19], as well as micro- and ultra-structural remodeling [20, 21]. Moreover, metabolomics of frozen fetal brain sections and *in vivo* brain metabolic profiling at birth indicate lower levels of mitochondrial tricarboxylic acid cycle (TCAc) metabolites, more prevalent in cortical regions [22, 23]. In fetal hearts, a higher distance between mitochondria and myofilaments, decreased mitochondrial density, and an overexpression of genes involved in cardiomyocyte oxidative metabolism have been reported [21]. How these basal structural and metabolic changes associated FGR actually affect the capacity of major organs to metabolize substrates, remains unknown. This information would improve our understanding about the functional effects of metabolic programming during FGR, and help tailor new therapeutic approaches *in utero*.

Here we investigate further the effects of FGR by assessing the ability of fetal brains and hearts to metabolize substrates as they become more available. Specifically, we infused fasted pregnant mothers with ^13^C-labeled substrates, such as glucose and acetate, and monitored FGR and appropriate for gestation age (AGA) fetal brains and hearts for differences in their products of cellular metabolism and phosphorylated intermediates, based on multinuclear High Resolution Magic Angle Spinning (HRMAS) of small tissue samples.

## Methods

### Animal model of FGR

All the experiments and animal procedures in this study were approved by the *Animal Experimental Ethics Committee* of the University of Barcelona (permit number: 458/16-9245). Pregnant New Zealand rabbits (Granja San Bernardo, Navarra, Spain) were housed at the animal facility of *Hospital Sant Joan de Déu*. At gestation day 25, FGR was induced in 9 rabbits, according to the protocol reported previously [23]. Prior to surgery, progesterone (0.9 mg/kg i.m.) and penicillin (G, 300,000 IU i.v.) were administered to each pregnant rabbit for tocolysis and prophylaxis, respectively. Anesthesia was induced by intramuscular injection of ketamine (35 mg/kg, *Ketolar*, Pfizer-Parke Davis S.L., Madrid Spain)–xylazine (5 mg/kg, *Rompun*, Bayer-KVP GmbH, Kiel, Germany) cocktail and maintained by endovenous perfusion (ketamina-xylazine: 1.87–0.56 mg/mL at 60 to 80 mL/h) on a v-shaped animal surgical table, where the animal is placed in supine position with respiratory support (oxygen mask: 4–5 liters / min) and a warming blanket in the back to maintain the body temperature. A local analgesic was administered along the abdominal midline (5 mL s.c. of 0.5% Bupivacaine, B.Braun, Barcelona, Spain), followed by laparotomy. Both uterine horns were exposed and the gestational sacs counted. One horn was returned to the abdominal cavity (AGA fetuses); in the remaining horn (FGR fetuses), the uteroplacental vessels of all gestational sacs were partially ligated (40–50%) using 4/0 silk sutures, while continuously rinsing with warm Ringer Lactate solution (Grifols Movaco S.A., Barcelona, Spain). The horn was then returned to the abdominal cavity, which was sutured in two layers (3/0 silk), and animals were left to recover under a warming blanket. Once animals became active, buprenorphine analgesia was administered subcutaneously (0.05 mg/kg Buprex, Indivior Ltd., Berkshire, UK), and they were returned to their cages. The same analgesia protocol was repeated every 24 h for 2 days, and pregnant rabbits were controlled daily. At gestational day 29, the pregnant rabbits were fasted overnight for 12 h with free access to water, and randomly assigned to 3 groups: 1-^13^C-glucose infusion (GLC, n = 4), 2-^13^C-acetate infusion (ACE, n = 3), and control, without infusion, (CTR, n = 2).

### Infusion of 1-^13^C-glucose

Lidocaine cream was applied over the rabbit ears 1 h before initiating the infusion procedure, under fasting conditions (gestational 30). The protocol for ^13^C-labeled glucose infusion (GLC group) was based on the method described by Habber *et al*. in pregnant New Zealand rabbits [24]. Thus, each rabbit was covered with a surgical cloth and left in a standard rat cage, to relieve stress and avoid sudden movements, respectively. The blood glucose level was measured with a glucometer (Contour XT, Bayer, Basel, Switzerland) before initiating the infusion, by fine puncture in the contralateral ear vein. Then, the marginal ear vein was cannulated with a 24 G catheter (Abbocath, Becton Dickinson, Utah, USA) and 1-^13^C-glucose (28 mM in saline) (99% 1-^13^C-glucose, Sigma-Aldrich, Schnelldorf, Germany) infused at a constant rate (0.2 mL · min^-1^ · kg^-1^) for 90 min. At 80 min of infusion, anesthesia was induced (ketamina-xylazine i.m., as before) and the animal transferred to the surgical table once asleep (90 min of infusion), where anesthesia, oxygen delivery and heating were performed as before. Infusion of 1-^13^C-glucose was maintained for another 20 min (375 mM in saline, same rate), while the animal was prepared for cesarean section. The mother’s blood glucose level was monitored once more at the end of the infusion (as before).

### Infusion of 2-^13^C-acetate

The protocol for ^13^C-acetate infusion was adapted from the method reported by Szczepaniak *et al*. in adult New Zealand rabbits [25]. In this case, the pregnant rabbit was anesthetized with ketamina-xylazine (35–5 mg/kg i.m.) and placed on the surgical table with oxygen delivery and heating, as described before. Once the animal was asleep, 2-^13^C-acetate (0.5M in saline; 99% 2-^13^C-acetate, Cambridge Isotopes, Andover MA, USA) was infused at a constant rate (0.14 mL · min^-1^ · kg^-1^) for 71 min, which was the time-to-steady-state of glutamate ^13^C-labiling according to [25]. Anesthesia was maintained with a half dose of the same cocktail (ketamina-xylazine 17.5–2.5 mg/kg i.m.), administered after 50 min of the initial dose (i.e. at 30 min of 2-^13^C-acetate infusion time).

### Sample collection

Fetal samples were collected at the cesarean section. Animals were already anesthetized in the ^13^C-subtrate infusion groups (GLC and ACE, after labeled-substrate infusion); whereas in the CTR group, anesthesia was induced and maintained with ketamina-xylazine, as described in the FGR induction section. Laparotomy was performed in all the animals and the AGA/FGR uterine horns identified to leave the more distal sacs outside the abdomen, perfused with warm ringer lactate solution. The fetuses were delivered one-by-one starting from the more distal sacs, and interleaved from the AGA and FGR horns. For consistency of the protocol, the first umbilical cord was always cut 10–13 min after the labeled-substrate infusion ended. After cutting the umbilical cord, the placenta was separated from the fetus, which was immediately weighted and decapitated to harvest samples. The latter step was performed in parallel by three individuals, and consisted in: (i) exposing the encephalon by inter-hemisferic sectioning with a scalpel and immersing the all head in liquid nitrogen; (ii) exposing the thoracic cavity to remove the heart and immerse it in liquid nitrogen; (iii) each sample piece was removed from the liquid nitrogen and temporarily stored in individual containers in dry ice, pre-cooled and pre-labeled. Only once steps (i) and (ii) were completed would sample harvesting from the next fetus begin. The time from cutting the umbilical cord to tissue sample immersions in liquid nitrogen was always <12 sec for each fetus. Once all the fetuses were delivered, the frozen samples were stored at -80°C until further use. At that point, the placentas were individually weighted and the anesthetized mother sacrificed with pentobarbital (200 mg/Kg *i*.*v*.).

### High Resolution Magic Angle Spinning (HRMAS)

The frozen tissue samples were analyzed *ex vivo* by High Resolution Magic Angle Spinning (HRMAS), at the joint NMR facility of *Universitat Autònoma de Barcelona* (SeRMN-UAB) and CIBER-BBN, Unit 25 of NANBIOSIS (www.nanbiosis.es). Thus, brain and heart samples were individually sectioned with a scalpel on dry ice to collect small pieces of cortical (inter-hemisferic frontal lobe) and apex tissues, respectively. The pieces were mounted in a cooled pre-weighted HRMAS rotor (Cortecnet, Paris, France) filled with 2 μL of D_2_O (99.9%, Carlo Erba Reactifs-SDS, Val de Reuil, France). The rotor was weighted before loading in the DMX 400 MHz spectrometer (Bruker BioSpin, Wissembourg, France), equipped with a ^1^H-^13^C/^31^P HRMAS resonance probe. All samples were studied in the same conditions (3000 Hz spinning rate and 4°C sample temperature) and with the same acquisition protocol (pre-set pulse calibrations and receiver gains, total duration 3.4 h), to enable the comparison of multinuclear 1D and 2D spectra from AGA and FGR subjects within each group. The HRMAS protocol consisted in a 10–25 min adjustment time (spin rate achieved, sample temperature stabilization, tuning/matching the probe channels, lock the D_2_O signal, slight shim adjustments) followed by consecutive acquisition of: 3 proton spectra (a ^1^H pulse-acquire non-suppressed water spectrum, a ^1^H pulse-acquire spectrum with water presaturation, and a ^1^H Carl-Purcell-Meiboom-Gill (CPMG) spectrum with water presaturation); 1 phosphorous spectrum (^1^H-decoupled ^31^P spectrum); changing the heteronuclear channel from ^31^P to ^13^C (2–5 min); 2 carbon spectra (a phase-sensitive 2D ^1^H-^13^C Heteronuclear Single-Quantum Correlation (HSQC) experiment, and a 1D ^1^H-decoupled ^13^C spectrum); and repeating the initial block of 3 proton spectra (19.3 min). Specific details for each sequence used, spectral processing and quantification procedures are included as S1 Materials.

### Statistical analyses

After confirming the normal distribution of the data within each group, with the Shapiro-Wilk test, statistical analyses were performed with a Student's t-test: two-tailed unpaired, when comparing between groups with different subjects in the same experimental conditions; and one-tailed paired, when comparing between groups with the same subjects at different time points. Results were considered significant at a p-value <0.05.

## Results

The most frequently used abbreviations are defined in **S1 Abbreviations**.

### Perinatal characteristics of the animal cohort

FGR was induced in 60 fetuses at 25 days of gestation, whereas 43 fetuses were counted in the AGA horns. As expected in this model, there was a higher stillbirth rate in the FGR horns (55%, n = 33) than in the AGAs (14%, n = 6) across the 3 experimental groups defined (CTR, GLC and ACE). The average birthweight of the live pups was also significantly lower in the former group (34.9±7.2 g) than in the latter (47.2±7.1 g, p<0.0001), as were the respective placental weights (4.5±1.5 vs. 5.6±1.3, p = 0.0053). Due to time restrictions for HRMAS access, a sub-cohort of FGR and AGA subjects was randomly selected. This included a total of 54 brain and heart tissue samples from 27 fetuses, with birthweights representative of the total cohort (Table 1). Thus, FGR fetuses had significantly lower birthweights than AGAs, in both GLC and ACE groups; but no significant differences were detected in either birthweight or placental weight between the different groups (CTR, GLC and ACE), when considering AGA and FGR fetuses separately (Table 1).

**Table 1:** Animal cohort used for HRMAS analyses.

Groups	Mothers	AGA Fetuses			FGR Fetuses		
	(n)	(n)	Birth Weight (g)	Placenta (g)	(n)	Birth Weight (g)	Placenta (g)
**CTR**	2	3	48.2±0.9	6.9±0.3	-	-	-
**GLC**	4	6	55.5±5.8	5.7±1.3	6	34.3±4.1 ^**a**^	4.3±1.7
**ACE**	3	6	51.0±2.4	5.1±2.0	6	33.1±5.5 ^**a**^	5.5±1.6

Numbers of mothers (total n = 9) and fetuses (n = 12 FGR and 15 AGA) used, and their average ±SD body and placental weights at birth for each group considered: CTR, GLC and ACE.

^**a**^ Unpaired Student’s t-Test (vs. AGA): p<0.0001.

### ^13^C-substrate infusions and metabolite labelling

**Fig 1:**
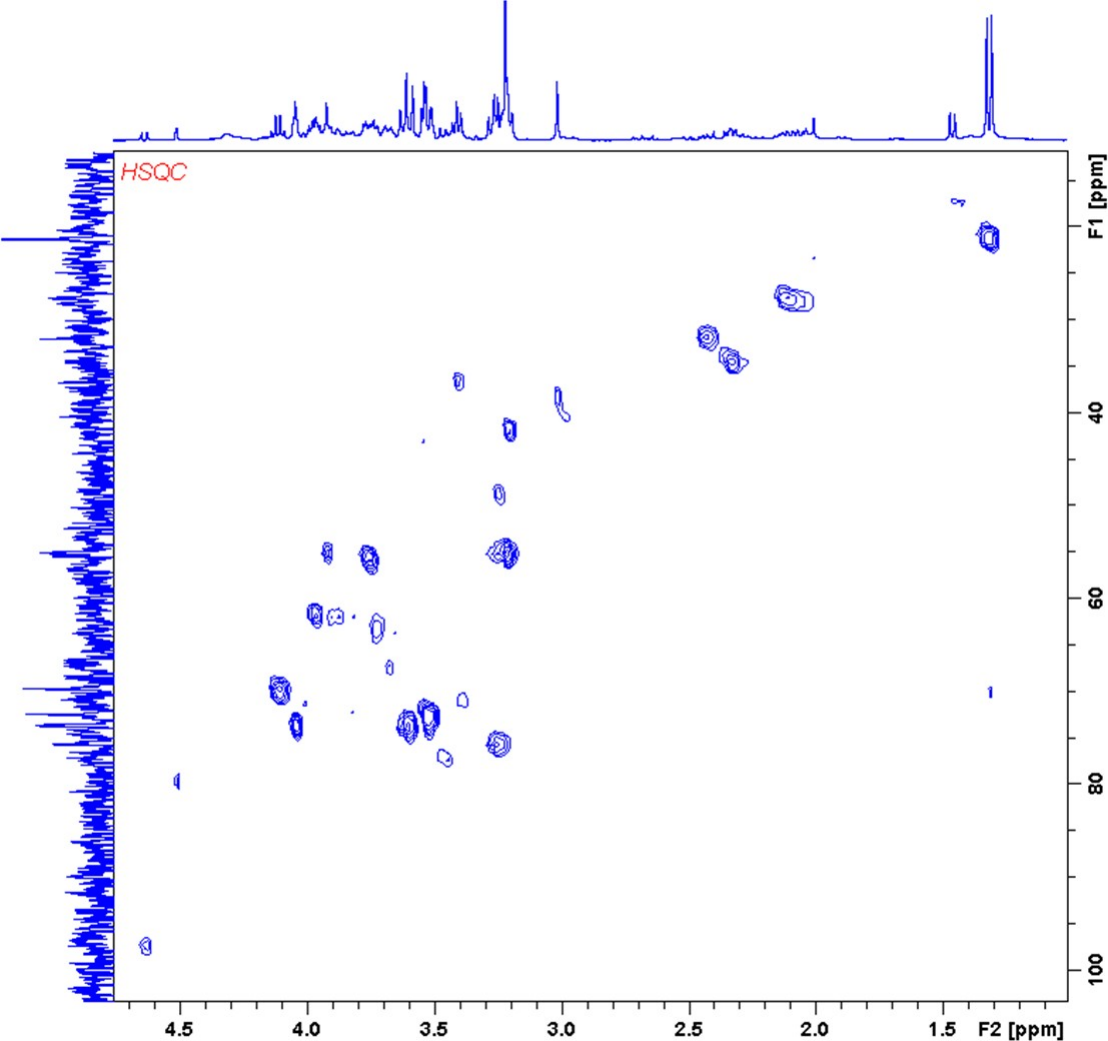
2D ^1^H-^13^C HSQC spectrum. Representative example from an AGA brain sample corresponding to the ACE group. Reference spectra displayed in the horizontal axis (^1^H CPMG) and vertical axis (^1^H-decoupled ^13^C), both referenced to lactate ^3^CH_3_ (^1^H, 1.32 ppm; ^13^C, 21.3 ppm). Spectrum zoomed to display only the major peaks and facilitate the interpretation of the results based on the assignments: lactate (Lac), glutamine (Gln) and glutamate (Glu). Remaining peak assignments displayed in S1 Fig.

A total of 7 rabbits were infused with ^13^C-labelled substrates: 4 rabbits with 1-^13^C-glucose, and 3 rabbits with 2-^13^C-acetate (Table 1). At the time of delivery, the mothers infused with 1-^13^C-glucose had increased their basal blood glucose levels by 2-fold (9.6±0.1 mM post-infusion vs. 5.1±0.4 mM basal). All tissue samples included in the study were prepared for HRMAS analyses according to the same protocol; 3 samples were discarded due to unexpected problems during loading in the spectrometer (rotor not inserting well in the probe and/or shimming problems). Accordingly, no significant differences were found in sample weight (49.7±3.0 mg, average±SD; constant D_2_O weight in each sample taken into account, 2.22 mg) between FGR-AGA samples in any group. Due to their low SNR, ^1^H-decoupled ^13^C spectra were used only to confirm assignments in the ^1^H-observed ^13^C spectra (2D ^1^H-^13^C HSQC), together with the ^1^H CPMG spectra (Fig 1). Thus, the high sensitivity of the 2D ^1^H-^13^C HSQC sequence enabled us to identify a total of 60 metabolite regions-of-interest (ROIs), which were reproduced as a template to quantify all the 2D spectra obtained (S1 Fig).

Our estimations of fetal ^13^C-subtrates incorporation and consumption indicate that about 90% of the total ^13^C-labeling reaching the brains (1-^13^C-glucose and 2-^13^C-acetate) and hearts (1-^13^C-glucose) was metabolized to alanine and lactate (cytosolic synthesis), and to glutamine and glutamate (mitochondrial synthesis) (Table 2). The relative incorporation of 2-^13^C-acetate to these metabolites was about 10% lower in the hearts, in which case the labeling of acetylcarnitine (3.3%), malate (2.7%) and pyruvate (2.5%) was slightly more pronounced. As expected, 2-^13^C-acetate was essentially consumed in the TCAc (78–86%), whereas 1-^13^C-glucose was mainly consumed in the cytoplasm through glycolysis (45–55% Lac C3’) and to a lower extent in the mitochondria via the TCAc (22–33%). Our estimations for the relative brain glucose fluxes through pyruvate dehydrogenase (PDH), pyruvate carboxylase (PC) and malic enzyme (ME) are shown in S2 Table. No significant differences between FGR and AGA samples were detected in any case (Table 2 and S2 Table). This is consistent with previous results with the same model, indicating no significant differences in umbilical artery pulsatility index at the time of delivery between FGR and AGA fetuses [18], and likely associated to a vasodilation effect consistently observed in the ligated horns at the time of delivery, as a possible compensation mechanism.

**Table 2:** Estimations of ^13^C-labelling incorporation in brain and heart metabolites.

^13^C-Intermediate	Total labelling incorporated (%)			
	GLC		ACE	
	Brain	Heart	Brain	Heart
**Ala C3**	12.9±1.5	13.4±2.8	3.6±3.5	1.7±1.0
**Lac C3**	56.5±8.6	45.7±18.5	12.1±5.6	3.7±2.8
**Lac C2**	0.9±1.9	0.5±2.8	11.1±5.6	2.4±2.1
**Gln C4**	1.0±0.9	1.1±0.9	14.6±2.7	5.4±1.9
**Glu C4**	10.8±4.0	11.6±7.8	17.0±6.3	14.2±4.0
**Glu C3**	3.8±2.4	13.3±6.6	15.7±4.5	32.6±2.7
**Glu C2**	5.0±2.1	6.3±5.5	16.7±5.6	21.3±2.6
**SUM (Ala+Lac+Glx)**	90.9±2.3	91.8±4.2	90.8±8.8	81.4±2.8
***Glycolysis (Lac C3')***	55.7±7.6	45.3±16.6	1.0±2.8	1.3±1.3
***TCAc (Glx+LacC2C3)***	22.4±6.5	33.2±15.7	86.3±11.7	78.4±3.0

Average ±SD values (% of total ^13^C-labelling) of pooled FGR and AGA fetuses for each tissue type and experimental group (GLC and ACE).

Abbreviations: Alanine (Ala), lactate (Lac), glutamine (Gln), glutamate (Glu), glutamate+glutamine pool (Glx), tricarboxylic acid cycle (TCAc).

Lac C3’ (synthesized from pyruvate C3, essentially derived from 1-^13^C-glucose) = Lac C3 –Lac C2; Lac C2C3 (synthesized from 1:1 pool of pyruvate C2 and C3, essentially derived from 1:1 pool of malate C2 and C3 shuttled from the mitochondria) = 2 · Lac C2.

### Differences between AGA and FGR subjects

#### ^13^C-labelled intermediates

**Fig 2:**
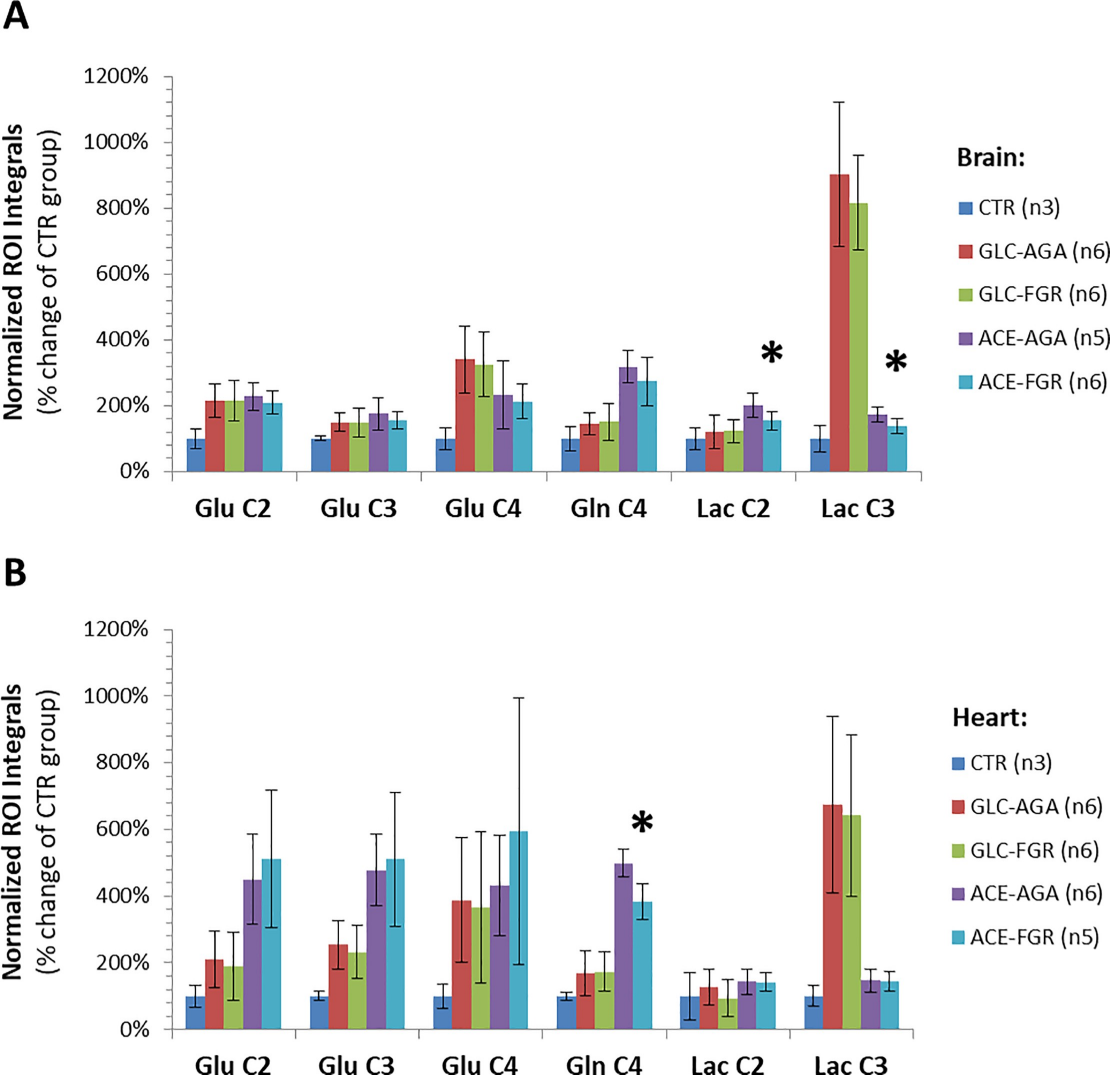
Quantification of ^13^C-enriched metabolite levels based on 2D ^1^H-^13^C HSQC spectra for different tissue samples and experimental groups. Values are displayed as normalized ROI integrals (average ±SD) for brain (**A**) and heart (**B**) samples of GLC and ACE groups, referenced to the CTR group (natural abundance of ^13^C, i.e. 1.1%). Metabolite labels as detailed in Fig 1 legend. Significant differences between AGA and FGR subjects are indicated (* p<0.05, two-tailed unpaired t-Test).

We then compared ^13^C-labeled metabolite levels between FGR and AGA samples, in all tissue types and experimental groups. No differences were detected in the GLC group, either for brain or heart samples. We only found significant differences in the ACE group. Thus, 2-^13^C-acetate-derived lactate had a slower turnover in FGR brains, as shown for the levels of Lac C2 (-23%, p = 0.0456) and Lac C3 (-20%, p = 0.0272) (Fig 2A), which have TCAc origin (S2 Fig) and are synthesized via ME. As for FGR hearts, we detected a significantly slower mitochondrial turnover of acetate-derived glutamine (Gln C4, -23%, p = 0.0029) (Fig 2B), consistent with an estimated tendency for lower GS activity (-36%, p = 0.070) (S3 Table).

Since the natural abundance of ^13^C is only 1.1% (i.e. metabolite levels measured in the CTR group), our results indicate that the ^13^C-enrichments in fetal brains and hearts were below 10% for all metabolites in the GLC and ACE groups (Fig 2). Thus, we additionally looked at the respective total levels of Lac and Gln in the ACE groups using ^1^H NMR, to assure that the differences in ^13^C-labeled substrates were due to differences in *de novo* synthesis rates during the infusions, rather than to changes in the respective basal levels (not turning over)–next section.

#### Corresponding basal metabolite pools (^1^H)

**Fig 3:**
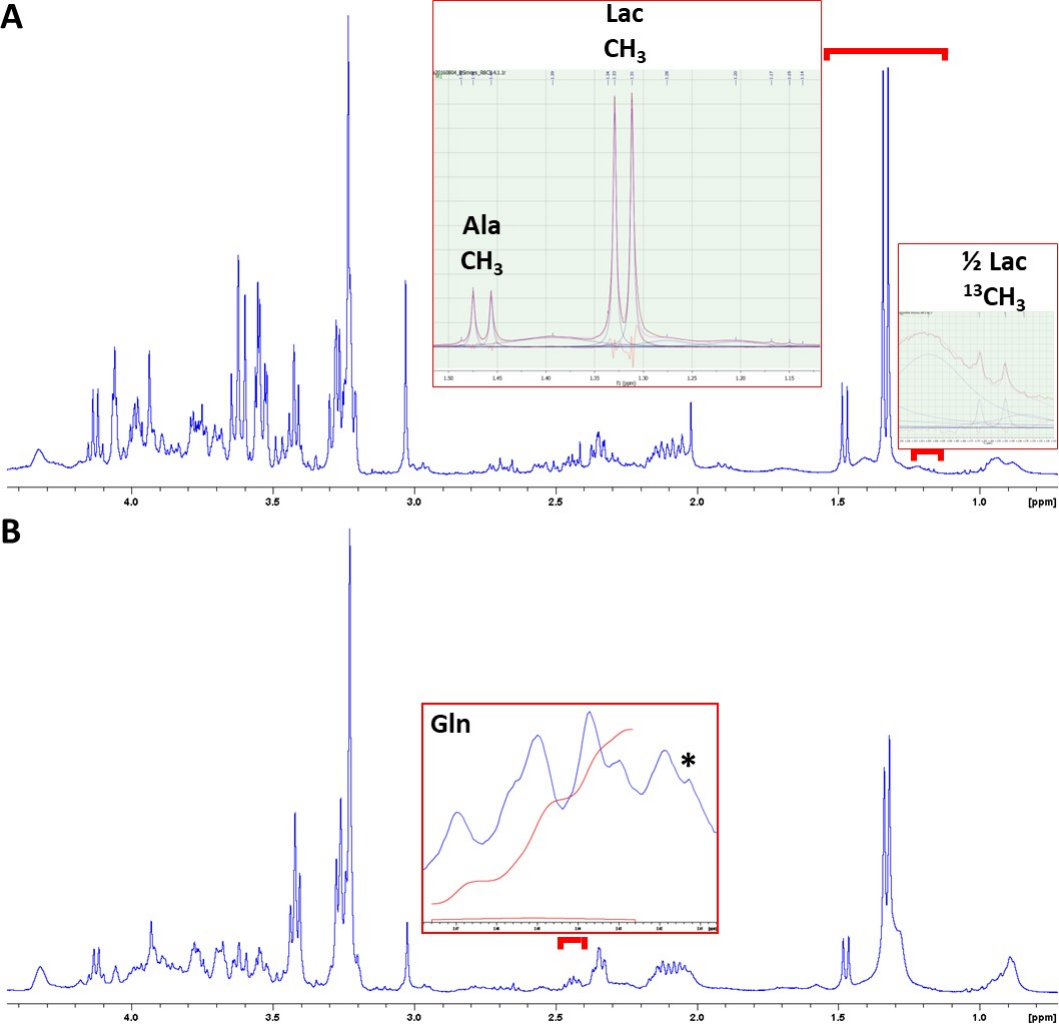
Initial ^1^H-CPMG spectra from brain and heart samples of the ACE group. Representative examples from the same animal subject, to display the quantification procedures for brain lactate (**A**) and heart glutamine (**B**). The CMPG filter did not fully saturate the lipid-macromolecule contributions to the spectral pattern, which were thus included in the 1.54–1.14 ppm deconvolution procedure (along with alanine CH_3_) for brain lactate quantification, both unlabeled (CH_3_ doublet, 1.32 ppm) and labeled satellite pools (two ^13^CH_3_ doublets around the CH_3_ doublet, with J-coupling 6.9 Hz–only quantified the one to the right, as displayed; the other one overlapping with Ala CH_3_) (**A**). The quantification of glutamine, with a clear multiplet pattern in heart samples (2.45 ppm), was performed instead by baseline-corrected integration over a 2.48–2.42 ppm spectral window, to discard the singlet contribution of succinate at 2.41 ppm (*) (**B**).

**Fig 4:**
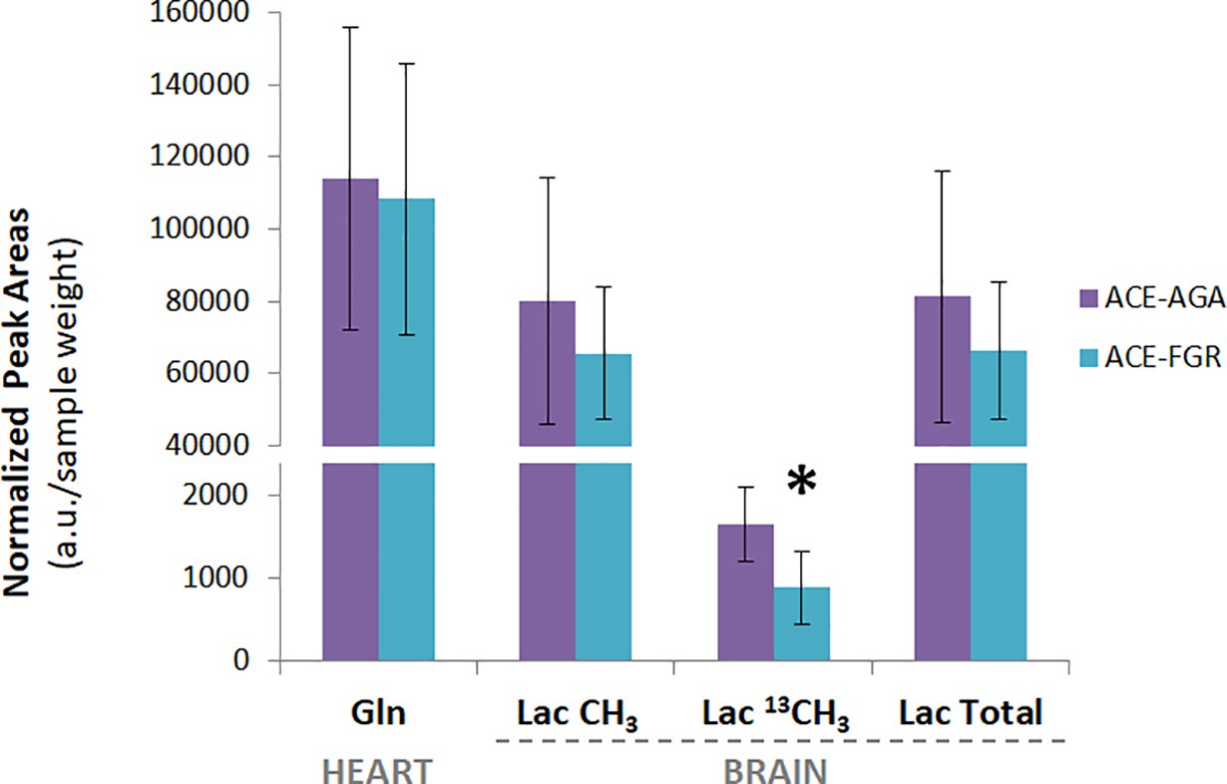
Quantification of heart glutamine and brain lactate pools of the ACE group. Values are displayed as metabolite peak areas normalized to sample weight (average ±SD), determined from ^1^H-CPMG spectra acquired at 34 min of HRMAS time (Fig 3A). Sample sizes (n): heart glutamine, 6 AGA and 5 FGR; brain lactate, 5 AGA and 6 FGR. Significant differences between FGR and AGA fetuses detected only for the estimated ^13^C-labelled lactate pool (* p<0.05, two-tailed unpaired t-Test), calculated as 2· ½ Lac ^13^CH_3._ Lac Total = Lac CH_3_ + Lac ^13^CH_3_.

We used the ^1^H-CPMG spectra acquired at the beginning of each HRMAS experiment to quantify ^3^CH_3_ lactate in heart samples (1.32 ppm, Fig 3A) and ^4^CH_2_ glutamine in brain samples (2.45 ppm, Fig 3B) of the ACE group. No significant differences were detected between AGA and FGR groups for total brain lactate (unlabeled + labeled pools) and total heart glutamine (Fig 4). However, the estimated labeled brain lactate levels (^13^CH_3_ satellites, difficult to quantify due to their low SNR) were significantly lower in the FGR group (p = 0.0377), in agreement with our 2D ^1^H-^13^C results for Lac C3 (Fig 2A).

Since time-course changes are also known to occur during HRMAS, we performed additional experiments (S1 Methods) and calculations to rule out the possibility of bias in the interpretation of our results (S1 Results).

#### Phosphorylated molecules (^31^P)

**Fig 5:**
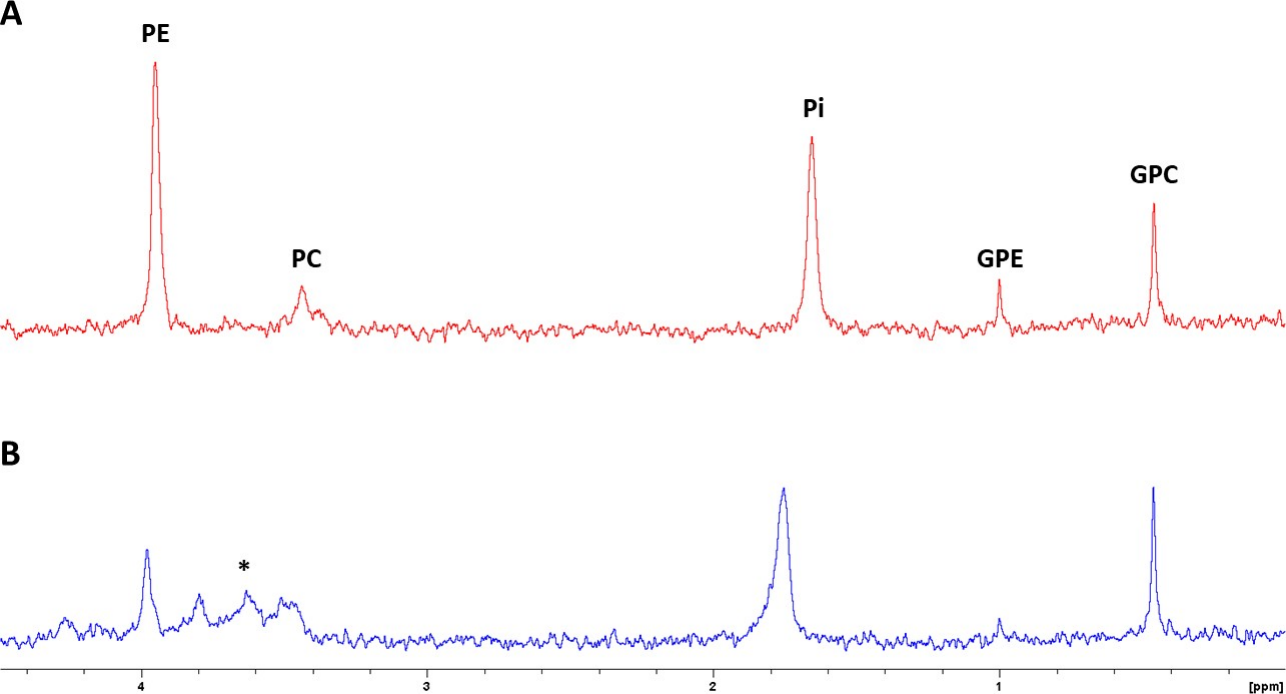
^1^H-decoupled ^31^P spectra. Representative examples from an AGA subject corresponding to the ACE group: brain (**A**) and heart (**B**) samples. Spectra referenced to glycerophosphoethanolamine (GPE, 1 ppm). Abbreviations: PE, phosphoethanolamine; PC, phosphocholine; GPC, glycerophosphocholine; Pi, inorganic phosphate. * region with additional peak contributions from 2,3-diphosphoglycerate, in the heart.

**Fig 6:**
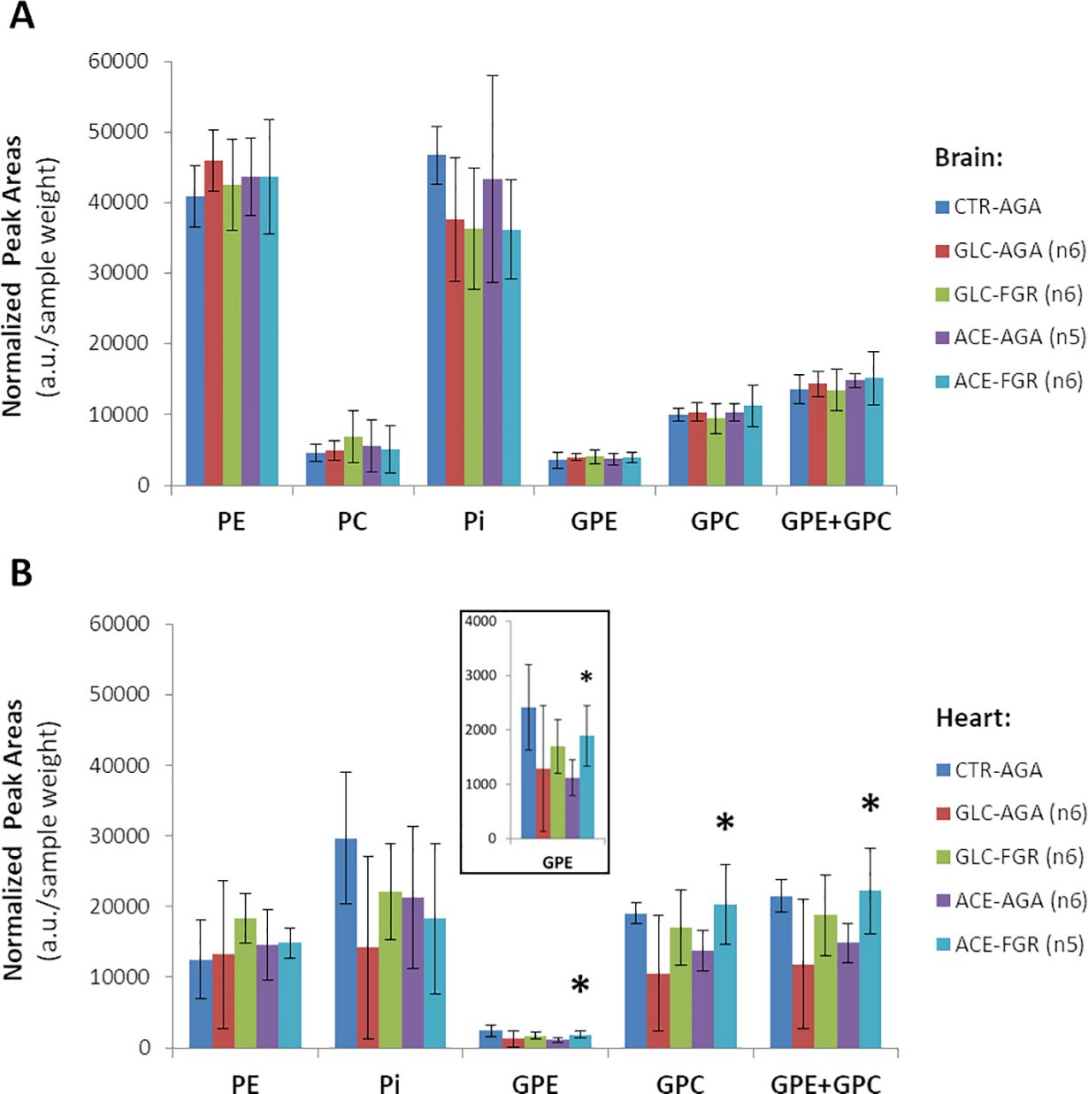
Quantification of phosphorylated metabolites based on ^31^P spectra for different tissue samples and experimental groups. Values displayed as metabolite peak areas normalized to sample weight (average ±SD) for brain (**A**) and heart (**B**) samples of CTR, GLC and ACE groups. PC could not be quantified properly in heart samples due to overlap with 2,3-diphosphoglycerate (Fig 5). Significant differences between AGA and FGR subjects are indicated (* p<0.05, two-tailed unpaired t-Test).

Based on ^1^H-decoupled ^31^P spectral data (Fig 5), no differences were detected in pH between FGR and AGA samples in GLC or ACE groups (brains, 6.44±0.08; hearts, 6.35±0.09). We only observed significant changes in heart phospholipid intermediates, specifically from the ACE group: higher accumulation of phosphodiesters (GPC and GPE) in FGR samples (+50% than in AGA), comparable to the CTR group profile; a similar tendency was observed in the GLC group (Fig 6).

## Conclusions

We have used a rabbit model and infusion of ^13^C-labelled substrates to assess the main functional changes in cerebral and cardiac metabolism of FGR compare to AGA fetuses, and potential differences in their cell membrane dynamics. No differences in ^13^C-labelled metabolite derivatives were detected following 1-^13^C-glucose infusion, only with 2-^13^C-acetate. Thus, *de novo* lactate (Lac C2C3, with TCAc origin) synthesis was slower in FGR brains, whereas the mitochondrial turnover of glutamine (Gln C4) was slower in FGR hearts, where a tendency for lower estimated GS activity was also noticed (accordingly). Moreover, phospholipid intermediates indicated a higher accumulation of phosphodiesters (GPC and GPE) in FGR compared to AGA samples, and a similar tendency was observed in the GLC group.

The number of stillbirths, birth weights and placental weights in each group were within the ranges previously reported for this model [14, 23]. In this work we were specifically interested in assessing the performance of fetal metabolism in the same cortical region previously studied *in vivo* at birth, to elucidate the metabolic background leading to the basal profile changes reported in FGR. At the same time we were interested in looking at the heart, given its well know susceptibility to placental insufficiency, encompassing structural and metabolic adaptations.

With regards to the infusion protocols, the original method described by Haber and Lapidot [24] was based on uniformly labeled glucose (synthesized in house), which leads to full labeling of the metabolites downstream of fructose 1,6-bisphosphate. Moreover, they performed metabolite extractions from large fetal brain samples (~500 mg each), which were pooled for *in vitro* high-resolution NMR analyses at slightly higher magnetic field strengths (500 MHz). Whereas in our case: (i) glucose was only labeled in the C1 position (only 50% labeling of *de novo* 3-carbon glycolytic metabolites and TCAc intermediates, such as lactate and the glutamine-glutamate pool, respectively); (ii) we used a 10-fold lower amount of tissue per sample (~50 mg); (iii) *ex vivo* analyses were performed from the fresh tissue, without an intermediate metabolite extraction/concentration step; and (iv), the samples were analyzed individually, from a single-tissue (cortex) and single-individual (not pooled). Thus, despite the high sensitivity of our 2D ^1^H-^13^C sequence we were not able to detect lower abundance metabolites, such as gamma-aminobutyric acid and Gln C2C3, as reported by others [24]. In any case, our results are in good agreement with the ^13^C-enrichments reported [24]: 5% enrichments in Glu C4 for normal fetal brains whereas we detected 2.4% for AGA brains (calculated as by [26], from the normalized ROI integrals for each isotopomer: (^13^C-Infused_GLC/ACE_ − Control_CTR_) / Control_CTR_), equivalent to 4.8% keeping in mind that our labelled metabolite pool is only 50% of [24]). Only Lac C3 labeling was about 2-fold higher in our case, i.e. 8% (equivalent to 16% total *de novo*) vs. 8.2%. This is likely explained by a slight difference in the sample collection protocol: while we froze the brains in liquid nitrogen after cutting umbilical cord, weighting, and decapitating the animal (<10 sec), Haber and Lapidot decapitated the fetuses prior to disconnection of the umbilical cord and froze the excised brains in liquid nitrogen directly. This should have saved them about 4–5 sec of *post-mortem* acute ischemia time (what we spent separating the placenta, cleaning membranes and weighting the animal).

**Fig 7:**
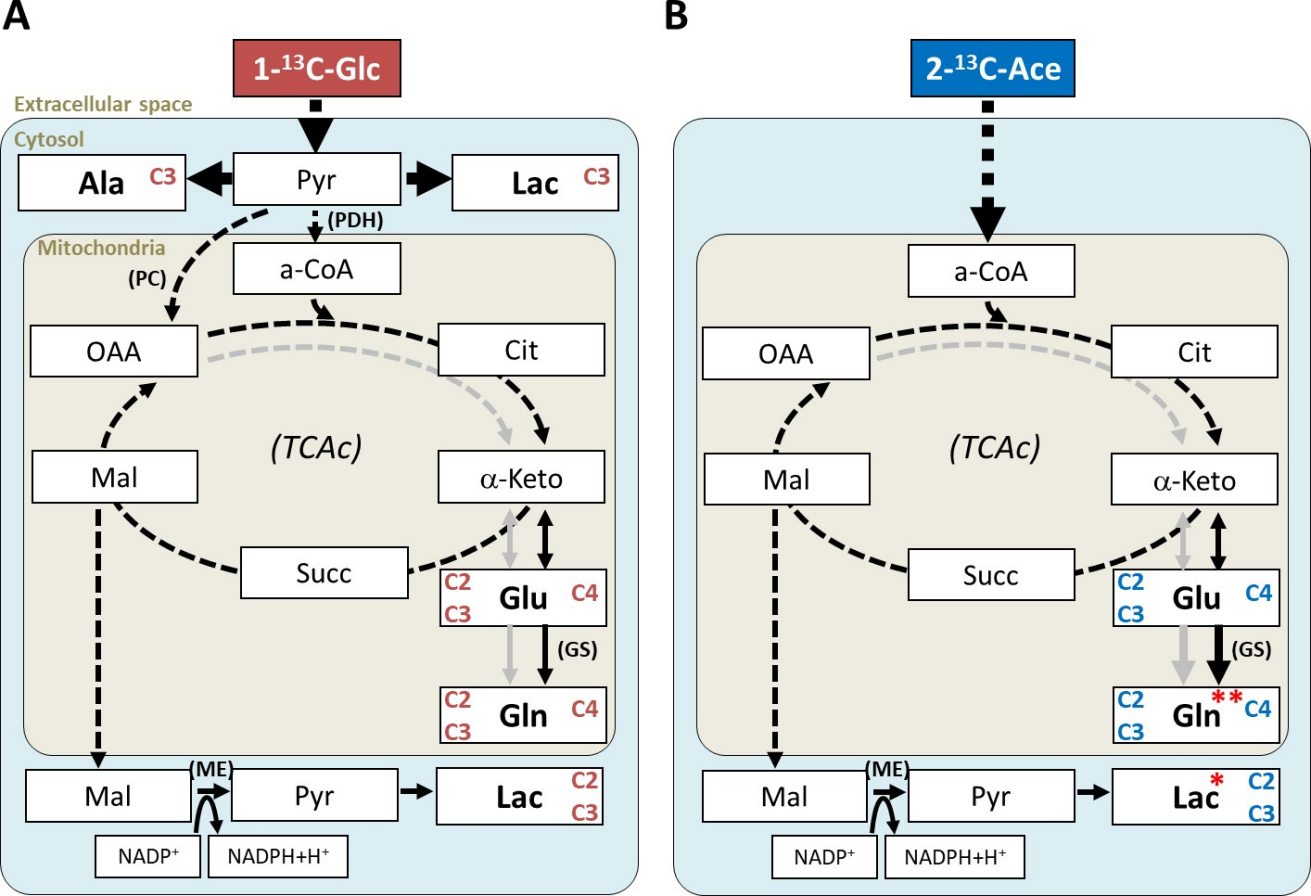
Models for the net cellular metabolic pathways of ^13^C-isotopomer intermediates in brains and hearts for each substrate infused. Each model represents the average metabolic pathways for the intermediates displayed, not accounting for intra-tissue heterogeneities (e.g. neurons vs. glia metabolic compartments), and according to infusions of: 1-^13^C-Glc **(A)** and 2-^13^C-Ace **(B)**. Labelled metabolite detectable in FGR and AGA fetal brain and heart samples are indicated with “Cn” (n = carbon position(s) for the labelling), and grey arrows indicate the second turn of the TCAc. Significant changes in FGR subjects: * brain, lower Lac C2C3 levels; ** heart, lower Gln C4 (and tendency for lower relative activity of GS). Other abbreviations: Cit, citrate; a-CoA, acetyl-coenzyme A; ɑ-Keto, alpha-ketoglutarate; Succ, succinate; Mal, malate; OAA, oxaloacetate; Pyr, pyruvate; PDH, pyruvate dehydrogenase; PC, pyruvate carboxylase; ME, malic enzyme; GS, glutamine synthetase.

**Models for the net cellular metabolic pathways of** ^**13**^**C-isotopomer intermediates** detected in each tissues and group are included in Fig 7, indicating the significant differences found. We did not detect any differences in metabolites derived from 1-^13^C-Glc, between FGR and AGA fetal brains or hearts. Since glucose labelling was mostly incorporated to glycolytic lactate and alanine synthesis (~ 50 and ~13%, respectively), these results are in agreement with our previous findings in fetal brains [22, 23], which did not indicate any basal changes in those metabolites between FGR and AGA subjects. Instead, those studies suggested differences in brain oxidative metabolism, associated with the mitochondrial TCAc. Our estimations of the relative brain glucose fluxes through PDH, PC and ME were within the ranges reported (PC/PDH = 0.14±0.04 in control rabbit fetuses [27]; PC/ME = 0.28±0.09 in neurons and 0.46±0.18 in astrocytes of primary cell cultures from embryo and neonatal rat brains, respectively [28]) but no significant differences were detected either between FGR and AGA brains.

TCAc metabolism was better assessed with 2-^13^C-Ace (~ 80% estimated labelling incorporation), since it can enter the TCAc directly through acetyl-CoA. Although the original protocol by Szczepaniak *et al*. [25] was reported in non-pregnant rabbits, and therefore no previous data were available for fetal tissues, the ^13^C-enrichments obtained were roughly within the same ranges obtained with 1-^13^C-Glc; except for Gln C4 and Lac C3, ≥2-fold higher in ACE and GLC groups, respectively. It should be stressed that while our 1-^13^C-Glc infusion protocol increased blood glucose by 2-fold, the estimated changes in blood acetate are nearly 10 times higher based on the literature: endogenous blood acetate in 2 kg adult male Japanese albino rabbits is ~0.21 mM [29], rising to ~3.7 mM after 70 min infusion in 3.5 kg male New Zealand rabbits [25]. Moreover, while glucose can be metabolized in the brain by both neurons and glia, acetate is preferentially metabolized in the latter to glutamine, due to glial-specific acetate uptake and GS activity [26, 30, 31].

Our results with **brain samples from fasted fetuses infused with acetate** suggest preferential glucose metabolism in neurons (Glu C4 >> Gln C4; PC/PDH = 0.2, due to glial-specific PC activity) and acetate in glia (Glu C4 < Gln C4), as expected. Specifically, we detected slower glial synthesis rate of lactate (Lac C2C3) in FGR brains than in AGA, suggests a glial impairment in the latter. This lactate pool has been described in fast proliferating cells with alternative TCAc fuels to glucose, as a mechanism to regenerate nicotinamide adenine dinucleotide phosphate (NADPH) reducing equivalents, to sustain cell proliferation [32]. A slower regeneration of NADPH (Fig 7) could in turn impact the maturation of glial cells, specifically oligodendrocytes. These cells are particularly sensitive to oxidative stress [33, 34] and NADPH is required to maintain cellular homeostasis of glutathione, a major antioxidant. NAPDH is also required for the biosynthesis of fatty acids and cholesterol, essential for myelination of white matter by oligodendrocytes [35]. Since myelination also relies on N-acetylaspartate (shuttled from neurons) as acetate donor, perhaps its lower levels in the FGR brains (cortex and hippocampus regions of this model [23], and in the frontal lobe of patients [5, 36]) do not represent the only cause leading to altered white matter microstructure [15], also observed in FGR infants [37].

With regards to **acetate-infused hearts**, we observed similar enrichments in all glutamate-glutamine isotopomers detected. In studies with isolated adult hearts perfused with 2- ^13^C-Ace, glutamine isotopomer levels have been measured and related to GS activity in mice [38], but mostly unreported in rabbits [39, 40]. It has also been shown that glutamine is primarily responsible for retaining ammonium in the rabbit myocardium [41]. Specifically, we detected a tendency for lower GS activity in fasted FGR subjects infused with labelled acetate compared to AGAs, in agreement with slower turnover of acetate to glutamine detected in the former. This slower mitochondrial turnover of glutamine through GS could imply a slower detoxification of ammonia from cardiomyocytes, as it accumulates e.g. during fasting periods [42]. Due to its role in the hexosamine biosynthesis pathway, glutamine is also implicated in cardiac fatty acid uptake, oxidation and storage [43], and cardioprotection (as mediator of glycosylation of nucleocytoplasmic proteins, by O-linked N-acetylglucosamine synthesis) under several stress conditions, such as ischemic injury [44, 45].

Moreover, our results suggested decreased cell membrane degradation in AGA hearts infused with acetate, compared with both infused FGR and non-infused control hearts (and similar tendency detected with glucose infusion). These changes were associated with higher levels of glycerophosphocholine (GPC) and glicerophosphoethanolamine (GPE) than in AGAs for the same FGR heart samples, but similar to the CTR group. Choline and ethanolamine phosphomonoesters (phosphocholine, PC, phosphoethanolamine, PE) and phosphodiesters (GPC and GPE) are particularly difficult to resolve in tissues by standard ^1^H MR spectroscopy due to their extensive overlap, and to our knowledge had not been assessed before in FGR fetal tissues. GPC and GPE are products of cell membrane degradation (phospholipid catabolism: phosphatidylcholine and phosphatidylethanolamine, respectively) and their accumulation has been extensively reported in several tissues as a response to cellular stress or disease, such as placentas with FGR [46, 47], cancer treatment [48], or brain of Alzheimer’s disease [49]. These observations suggest that FGR fetuses are less amenable to adapting their cardiac metabolism in response to increasing substrate availability, in order to sustain and/or improve cellular growth. This complements our previous knowledge in this animal model of FGR (decreased number of cardiomyocytes [20], with lower mitochondrial density and altered energetic metabolism [21]) and may contribute to an increased susceptibility to cardiac disease, which has long been observed clinically [13].

The strengths of this work rely in a well characterized animal model that reproduces many clinical features of FGR. This study improves substantially our knowledge about the metabolic programming in this model, since all previous studies relied on basal profiles of cerebral metabolism and cardiac gene expression, not on their functional metabolic responses. This was achieved using non-radioactive isotope-labelling (^13^C) and statistical significances were reached with a relatively small animal cohort and a scrupulous analysis of the data, to prevent biased interpretations of the results.

Some limitations should be acknowledged. We did not measure blood pressure or oxygenation during the experiments, which together with glucose concentration changes and the effects of anesthetics and analgesics, are expected to alter cerebral and cardiac metabolism [50–53]. This may raise concern about the pertinence of our findings for characterizing the metabolic effects of FGR in non-anesthetized animals; however, the relative differences reported between AGA and FGR groups are relevant since both were kept under the same physiologic conditions. Then, since sacrifice by focus microwave irradiation [54] was not available, we must address two potential sources of bias in our results: post-mortem accumulation of lactate, and metabolite time-course changes during HRMAS. While lactate effects should be expected to equality impact FGR and AGA fetuses, HRMAS effects are well reported and were controlled in each HRMAS experiment, and rigorously accounted for when analyzing the results (S1 Results). Finally, we were not able to quantify phosphocholine in heart samples due to the interference of 2,3-diphosphoglycerate, as reported by others [55].

To conclude, FGR fetuses present specific functional differences in cerebral and cardiac metabolism. This suggests that FGR tissues could be less adaptable to microenvironmental changes associated with perinatal stress conditions, potentially underlying the neurodevelopmental and cardiovascular problems previously observed in this animal model and in FGR patients. Follow up studies should evaluate how these metabolic changes persist in adult life, specifically in response to cellular stress and/or prenatal treatment strategies to ameliorate outcome [56].

## Supporting information

S1 Abbreviations List of abbreviations. (DOCX)

S1 Methods Supplementary methods file. (DOCX)

S1 Results Supplementary results file. (DOCX)

S1 References Supplementary references file. (DOCX)

S1 Table HRMAS sequences. Acquisition parameters specified for each sequence, including excitation pulse (p1), number of points (Points), number of scans (Scans) and total acquisition time (Time).
(DOCX)

S2 Table Relative glucose fluxes in brain samples. Calculated as detailed in S1 Methods section and based on Fig 7A model. No significant differences detected between FGR and AGA samples. Abbreviations: PC, pyruvate carboxylase (glial-specific); PDH, pyruvate dehydrogenase; ME, malic enzyme.
(DOCX)

S3 Table Estimation of GS relative activity based on ^13^C-isotopomer ratios. Average values ±SD are displayed for FGR and SGA subjects according to tissue type (brains and hearts) and study group (GLC and ACE).
(DOCX)

S1 Fig ROIs for quantification of 2D ^1^H-^13^C HSQC spectra. A total of 60 metabolite peaks (and 4 noise regions) were included in the template (A), quantified by integration and normalized to sample weight for brain (B) and heart (C) tissues.
(DOCX)

S2 Fig Estimations of brain ^13^C-lactate enrichments from glycolysis and TCAc. Values are displayed as normalized ROI integrals (average ±SD) for GLC and ACE groups, referenced to the CTR group (%). Significant differences between AGA and FGR subjects are indicated (* p<0.05, two-tailed unpaired t-Test). Lac C3’ (synthesized from pyruvate C3, essentially derived from 1-13C-glucose) = Lac C3 –Lac C2; Lac C2C3 (synthesized from 1:1 pool of pyruvate C2 and C3, essentially derived from 1:1 pool of malate C2 and C3 shuttled from the mitochondria) = 2 · Lac C2.
(DOCX)

S3 Fig Time-course changes detectable in 2D ^1^H-^13^C spectra. Brain (A) and heart (B) tissues resampled from one of the AGA-ACE subjects used for the main experiments. Linear adjustments for Gln C4 (blue), Lac C2 (red) and Lac C3 (green) confirmed a slight accumulation of these metabolites over 5.2 h.
(DOCX)

S4 Fig Quantification of heart glutamine and brain lactate pools based on the final ^1^H-CPMG spectra of the ACE group. Values based on the final ^1^H-CPMG spectrum acquired in each HRMAS session (**A**), indicating metabolite peak areas normalized to sample weight (average ±SD). Significant differences between FGR and AGA fetuses detected only for the estimated ^13^C-labelled lactate pool, ^13^CH_3_ (* p<0.05, two-tailed unpaired t-Test)_._ Difference between the metabolite quantifications in A and the respective levels obtained from the initial ^1^H-CPMG sequence (Fig 4) (**B**). Sample sizes (n): heart glutamine, 6 AGA and 5 FGR; brain lactate, 5 AGA and 6 FGR. Lac ^13^CH_3_ = 2· ½ Lac ^13^CH_3_; Lac Total = Lac CH_3_ + Lac ^13^CH_3_.
(DOCX)
