# Supplementary material for: Assessment of prenatal cerebral and cardiac metabolic changes in a rabbit model of fetal growth restriction based on 13C-labelled substrate infusions and ex vivo multinuclear HRMAS
Source: PLoS One. 2018 Dec 27;13(12):e0208784. doi: 10.1371/journal.pone.0208784 (PMC6307735; doi:10.1371/journal.pone.0208784)
Supplement: S1 Abbreviations — (DOCX) [file pone.0208784.s001.docx]

**S1 Abbreviations**

**AGA** appropriate for gestational age

**CTR** control

**FGR** fetal growth restriction

**HRMAS** high resolution magic angle spinning

**Glc** glucose

**Gln** glutamine

**GPC** glycerophosphocholine

**GPE** glycerophosphoethanolamine

**Lac** lactate

**ME** malic enzyme

**NMR** nuclear magnetic resonance

**PC** pyruvate carboxylase

**PDH** pyruvate dehydrogenase

**TCAc** tricarboxylic acid cycle
