# Supplementary material for: Assessment of prenatal cerebral and cardiac metabolic changes in a rabbit model of fetal growth restriction based on 13C-labelled substrate infusions and ex vivo multinuclear HRMAS
Source: PLoS One. 2018 Dec 27;13(12):e0208784. doi: 10.1371/journal.pone.0208784 (PMC6307735; doi:10.1371/journal.pone.0208784)
Supplement: S1 Methods — (DOCX) [file pone.0208784.s002.docx]

**S1 Methods**

**1. HRMAS data acquisition**

The specific parameters for each HRMAS sequence used are listed in S1 Table. An additional experiment was performed to evaluate time-course 2D ^1^H-^13^C changes. The rotors were mounted with fresh tissue samples (not the ones used before) and the HRMAS protocol (5.2 h total time) consisted of: 15 min adjustment time (as before), two consecutive 2D ^1^H-^13^C HSQC acquisitions, a 1D ^1^H-decoupled ^13^C acquisition, a ^1^H-CPMG acquisition, and another 2D ^1^H-^13^C HSQC acquisition; all the sequences with the same parameters described before (S1 Table).

**2. Spectral processing and quantification**

The HRMAS data were processed (phase correction and line broadening to: 0.3 Hz in ^1^H spectra, and 1 Hz in ^31^P and ^13^C spectra) and metabolite peak regions were quantified either by deconvolution (fitting baseline-corrected ^1^H CPMG and ^31^P spectra, *MestReNova* 11.0 software, *MestreLab Research S.L.* Santiago de Compostela, Spain) or integration (*TopSpin* 3.5 software, Bruker BioSpin Rheinstetten, Germany): using automated baseline-correction for ^1^H CPMG spectra; and baseline-correction with degree 5 polynomial least square fit (*qpol* function) to subtract the strong water signal from the 2D ^1^H-^13^C spectra before defining the metabolite regions-of-interest (ROIs). In the latter spectra, 4 ROIs corresponding to noise areas were also included (similar geometry as the metabolite ROIs), to help discarding signals from further analyses. The metabolite quantifications were then normalized to sample weight (in the HRMAS rotor) for further analyses. Additionally, the pH of the samples was calculated as reported previously in cell lines **[1^supp^]**, based on the chemical shift of the inorganic phosphate peak (Pi) relative to the glycerophosphoethanolamine (GPE) peak calibrated to 1 ppm:

pH = 6.66 + LOG10((δPi-0.729)/(3.22-δPi)) **(Eq. 1)**

**3. Analyses of 2D ^1^H-^13^C** **data**

Since the 2D ^1^H-^13^C spectra from the CTR group showed basal levels for most metabolites detectable, their quantification results were analyzed in two ways to investigate *de novo* synthesis. Firstly, to estimate the ^13^C-labelling incorporation in brain and heart metabolites of the GLC and ACE groups. Thus, the average basal levels for each metabolite in the CTR group were individually subtracted from the respective measurements in the FGR and AGA samples from the other groups, to estimate the *de novo* levels for each metabolite, i.e. synthesized during the ^13^C-substrate infusions. Metabolite peaks without evident *de novo* synthesis in the different samples for each tissue and study group were discarded; the rest were normalized to their sum and displayed as percentage of total. The FGR and AGA results were then pooled for each tissue (brain and heart) and experimental group (GLC and ACE). Secondly, the 2D ^1^H-^13^C normalized quantifications were used to determine differences in ^13^C- metabolite enrichments between FGR and AGA samples in each group (GLC and ACE). In this case, the CTR group was used as reference (%), in order to reduce potential biases in the group comparison due to data manipulation. The latter approach was additionally used for estimations based on Fig. 7 models: (i) relative brain glucose fluxes through pyruvate dehydrogenase (PDH), pyruvate carboxylase (PC) and malic enzyme (ME), as reported by others (PDH = Glu C4; PC = Glu C2 – Glu C3; ME = 2x Lac C2; all estimations based on normalization to total flux, PDH + PC + ME =1); and (ii), relative enzymatic activity of glutamine synthetase (GS) in all tissues and groups studied, based on the Gln C4 (product) to Glu C4 (substrate) ratio.
