# Supplementary material for: Assessment of prenatal cerebral and cardiac metabolic changes in a rabbit model of fetal growth restriction based on 13C-labelled substrate infusions and ex vivo multinuclear HRMAS
Source: PLoS One. 2018 Dec 27;13(12):e0208784. doi: 10.1371/journal.pone.0208784 (PMC6307735; doi:10.1371/journal.pone.0208784)
Supplement: S1 Results — (DOCX) [file pone.0208784.s003.docx]

**S1 Results**

Metabolite time-course changes have been reported during long ^1^H-HRMAS experiments, even at low temperature **[5-7^supp^]**. Thus, the metabolite changes detected by 2D ^1^H-^13^C (de novo Gln and Lac) were of particular concern, since the acquisition started 50 min after the samples were loaded in the spectrometer, and lasted 79 min. Thus, we considered this a potential source of bias for the interpretation of our results and performed additional experiments and calculations to rule it out.

First, we run two time-course 2D ^1^H-^13^C experiments (S1 Methods**)**, for brain and heart tissues resampled from one of the AGA-ACE subjects (used previously for the main experiments). This confirmed a slight accumulation of Gln C4, Lac C2, and Lac C3 over 5.2 h of HRMAS, which was already detectable after 2.9 h (S3 Fig).

Then, we used the ^1^H-CPMG spectra acquired at the end of each HRMAS experiment in the ACE group, to quantify ^3^CH_3_ lactate in heart samples (1.32 ppm, both unlabeled and labeled satellite pools – as shown in 3A Fig) and ^4^CH_2_ glutamine in brain samples (2.45 ppm, total pool - as shown in Fig 3B) and compare them with the respective levels at the beginning of the HRMAS experiment (Fig 4**)**. Total brain lactate (unlabeled + labeled) and total heart glutamine increased over time, as expected (S3 Fig), but no significant differences between AGA and FGR groups were observed (S4A Fig), or for the respective time-course changes (S4B Fig). Only the estimated labeled brain lactate levels (^13^CH_3_ satellites) remained significantly lower in the FGR group (p = 0.0194 – S4A Fig); the respective time-course changes were also not significantly different between AGA and FGR groups (S4B Fig).

In addition, we analyzed the initial adjustment times (i.e. time from sample loading in the spectrometer to data acquisition, 16±8 min), to investigate if this parameter could potentially have influenced our metabolite quantifications in the ACE group, according to S3 Fig. We found no positive correlations between initial adjustment times and normalized levels of brain lactate C2 and C3, or heart glutamine C4, as determined from 2D ^1^H-^13^C spectra (S1 Fig); or the respective total metabolite pools detected by ^1^H-CPMG. However, we found a tendency for shorter adjustment times in FGR heart samples than in AGA (average±SD: 11±1 vs. 17±5 min, p=0.0588); not in brain samples. Since we could not rule out the latter observation as a potential source of bias, we then compared the FGR group with a subset of AGA heart samples (n=3) within the same adjustment time ranges (average±SD: 11±1 vs. 13±4 min, p=0.4133). Thus, glutamine C4 remained significantly lower in the FGR group (-24%, p=0.0126) while no changes were detected in total glutamine between the two groups.

With regards to the phosphorylated intermediates GPC and GPE (differences detected between FGR and AGA hearts), based on the literature **[6-8^supp^]** we did not expect significant time-course changes since ^31^P spectra were acquired at an earlier time-point (35min of HRMAS). Specifically, Payne *et al.* did not detect significant changes in GPC, GPE, PE or PC during 100 min of ^31^P-HRMAS at 4 °C, with samples also spinning at 3kHz **[8^supp^]**. Based on ^1^H-HRMAS, Swanson *et al.* reported a ~3% linear decrease of GPC+PC at 100 min of ^1^H-HRMAS at 1 °C, using a 2.25kHz spinning rate (-10% after 6.5 h) **[3^supp^]**, whereas Opstad *et al.* evaluated the effect of ≥30min ischemia time on ^1^H-HRMAS time-course changes of GPC, with the sample spinning at 5kHz **[4^supp^]**. Since our protocol was intermediate (4-8 sec ischemia period, between animal sacrifice and sample freezing in liquid nitrogen), we expected only minor changes in the first 30min, if any. In any case, using the previous subset of AGA heart samples (n=3) as reference still indicated tendencies for higher GPC and GPE levels in the FGR group: +63% GPC (p=0.0670); and +68% GPE (p=0.0668).

These additional data support our interpretation, i.e. the differences in the *de novo* pools of brain lactate and heart glutamine from the ACE group should be associated with prenatal metabolic changes due to FGR; not to *post-mortem* effects taking place during the HRMAS experiments. The latter are expected to have only a minor effect, if any.
