# Supplementary material for: Assessment of prenatal cerebral and cardiac metabolic changes in a rabbit model of fetal growth restriction based on 13C-labelled substrate infusions and ex vivo multinuclear HRMAS
Source: PLoS One. 2018 Dec 27;13(12):e0208784. doi: 10.1371/journal.pone.0208784 (PMC6307735; doi:10.1371/journal.pone.0208784)
Supplement: S1 References — (DOCX) [file pone.0208784.s004.docx]

**S1 References**

# 1^supp^. Simões RV, Serganova IS, Kruchevsky N, Leftin A, Shestov AA, Thaler HT, et al. (2015) Metabolic plasticity of metastatic breast cancer cells: adaptation to changes in the microenvironment. Neoplasia 17: 671-684.

**2^supp^.** Lapidot A, Haber S (2000) Effect of acute insulin-induced hypoglycemia on fetal versus adult brain fuel utilization, assessed by (13)C MRS isotopomer analysis of [U-(13)C]glucose metabolites. Dev Neurosci 22: 444-455.

**3^supp^.** Brand A, Richter-Landsberg C, Leibfritz D (1993) Multinuclear NMR studies on the energy metabolism of glial and neuronal cells. Dev Neurosci 15: 289-298.

**4^supp^.** Simao D, Terrasso AP, Teixeira AP, Brito C, Sonnewald U, Alves PM. (2016) Functional metabolic interactions of human neuron-astrocyte 3D in vitro networks. Sci Rep 6: 33285.

**5^supp^.** Cheng LL, Ma MJ, Becerra L, Ptak T, Tracey I, Lackner A, et al. (1997) Quantitative neuropathology by high resolution magic angle spinning proton magnetic resonance spectroscopy. *Proc. Natl. Acad. Sci.* 94: 6408–6413.

**6^supp^.** Swanson MG, Zektzer AS, Tabatabai ZL, Simko J, Jarso S, Keshari KR, et al. (2006) Quantitative Analysis of Prostate Metabolites Using ^1^H HR-MAS Spectroscopy. Magn Reson Med 55:1257–1264.

**7^supp^.** Opstad KS, Bell BA, Griffiths JR, Howe FA (2008) An assessment of the effects of sample ischaemia and spinning time on the metabolic profile of brain tumour biopsy specimens as determined by high-resolution magic angle spinning ^1^H NMR. NMR Biomed 21: 1138–1147.

**8^supp^.** Payne GS, Troy H, Vaidya SJ, Griffiths JR, Leach MO, Chung YL. (2006) Evaluation of ^31^P high-resolution magic angle spinning of intact tissue samples. NMR Biomed 19:593-598.
