# Supplementary material for: Assessment of prenatal cerebral and cardiac metabolic changes in a rabbit model of fetal growth restriction based on 13C-labelled substrate infusions and ex vivo multinuclear HRMAS
Source: PLoS One. 2018 Dec 27;13(12):e0208784. doi: 10.1371/journal.pone.0208784 (PMC6307735; doi:10.1371/journal.pone.0208784)
Supplement: S1 Table — Acquisition parameters specified for each sequence, including excitation pulse (p1), number of points (Points), number of scans (Scans) and total acquisition time (Time). (DOCX) [file pone.0208784.s005.docx]

**S1 Table. HRMAS sequences**.

| **Sequence** | **Nucleus** | **p1**  **(µs)** | **Relaxation delay (s)** | **Spectral Width (Hz)** | **Points (#)** | **Scans (#)** | **Time (min)** |
| --- | --- | --- | --- | --- | --- | --- | --- |
| *zg* | ^1^H | 7 | 8 | 4,000 | 16k | 8 | 1.7 |
| *zgpr* | ^1^H | 7 | 2 | 4,000 | 16k | 128 | 8.8 |
| *cpmgpr* | ^1^H | 7 ***^a^*** | 2 | 4,000 | 16k | 128 | 8.8 |
| *zgpg60* ***^b^*** | ^31^P | 10.5 | 0.5 | 1,965 | 16k | 128 | 10.2 |
| *hsqcetgspi* ***^c^*** | ^13^C | 7 ***^d^*** | 1 | 4,000 ***^e^*** | 1k ***^f^*** | 32 | 79 |
| *zgpg60d* ***^g^*** | ^13^C | 10.5 | 6 | 29,762 | 16k | 512 | 53.6 |

Acquisition parameters: excitation pulse (p1), number of points (Points), number of scans (Scans) and total acquisition time (Time).

***^a^*** All pulses: p1 (90° ^1^H), 7 µs; p2 (180° ^1^H).

***^b^*** ^1^H-decoupled, 60° pulse-acquire ^31^P.

***^c^*** phase-sensitive 2D ^1^H-^13^C Heteronuclear Single-Quantum Correlation (HSQC).

***^d^*** All pulses: p1 (90° ^1^H), 7 µs; p2 (180° ^1^H), 14 µs; p3 (90° ^13^C), 10.5 µs; p4 (180° ^13^C), 21 µs.

***^e^*** ^1^H dimension; 22,136 Hz in the ^13^C dimension.

***^f^*** Number of increments: 128.

***^g^*** ^1^H-decoupled, 60° pulse-acquire ^13^C.
