# Supplementary material for: Assessment of prenatal cerebral and cardiac metabolic changes in a rabbit model of fetal growth restriction based on 13C-labelled substrate infusions and ex vivo multinuclear HRMAS
Source: PLoS One. 2018 Dec 27;13(12):e0208784. doi: 10.1371/journal.pone.0208784 (PMC6307735; doi:10.1371/journal.pone.0208784)
Supplement: S2 Table — Calculated as detailed in S1 Methods section and based on Fig 7A model. No significant differences detected between FGR and AGA samples. Abbreviations: PC, pyruvate carboxylase (glial-specific); PDH, pyruvate dehydrogenase; ME, malic enzyme. (DOCX) [file pone.0208784.s006.docx]

**S2 Table. Relative glucose fluxes in brain samples.**

| . **Enzymes**  **(metabolic pathways)** | **Relative glucose fluxes ^a^** | |
| --- | --- | --- |
|  | **AGA** | **FGR** |
| **PC/PDH** | 0.19±0.06 | 0.21±0.06 |
| **PC/ME** | 0.30±0.14 | 0.31±0.19 |
| **PDH** | 0.53±0.12 | 0.50±0.11 |

Calculated as detailed in S1 Methods section and based on Fig. 7A model; no significant differences detected between FGR and AGA samples.

Abbreviations: PC, pyruvate carboxylase (glial-specific); PDH, pyruvate dehydrogenase; ME, malic enzyme.
