# Supplementary material for: Assessment of prenatal cerebral and cardiac metabolic changes in a rabbit model of fetal growth restriction based on 13C-labelled substrate infusions and ex vivo multinuclear HRMAS
Source: PLoS One. 2018 Dec 27;13(12):e0208784. doi: 10.1371/journal.pone.0208784 (PMC6307735; doi:10.1371/journal.pone.0208784)
Supplement: S3 Table — Average values ±SD are displayed for FGR and SGA subjects according to tissue type (brains and hearts) and study group (GLC and ACE). (DOCX) [file pone.0208784.s007.docx]

**S3 Table. Estimation of GS relative activity based on ^13^C-isotopomer ratios**.

| **Enzyme** | **Relative activity ^a^** | | | | | | | |
| --- | --- | --- | --- | --- | --- | --- | --- | --- |
|  | **GLC** | | | | **ACE** | | | |
|  | **Brain** | | **Heart** | | **Brain** | | **Heart** | |
|  | **AGA** | **FGR** | **AGA** | **FGR** | **AGA** | **FGR** | **AGA** | **FGR** |
| **GS** | .44±.11 | .49±.18 | .46±1.17 | .63±.39 | 1.47±.32 | 1.30±.22 | 1.27±.40 | 0.81±.32 **^b^** |

Average values ±SD displayed for FGR and SGA subjects according to tissue type (brains and hearts) and study group (GLC and ACE).

**^a^** =Gln C4 /Glu C4;

**^b^** Unpaired Student’s t-Test (vs. AGA): p=0.070.
