# Supplementary material for: Assessment of prenatal cerebral and cardiac metabolic changes in a rabbit model of fetal growth restriction based on 13C-labelled substrate infusions and ex vivo multinuclear HRMAS
Source: PLoS One. 2018 Dec 27;13(12):e0208784. doi: 10.1371/journal.pone.0208784 (PMC6307735; doi:10.1371/journal.pone.0208784)
Supplement: S1 Fig — A total of 60 metabolite peaks (and 4 noise regions) were included in the template (A), quantified by integration and normalized to sample weight for brain (B) and heart (C) tissues. (DOCX) [file pone.0208784.s008.docx]

**A**

**
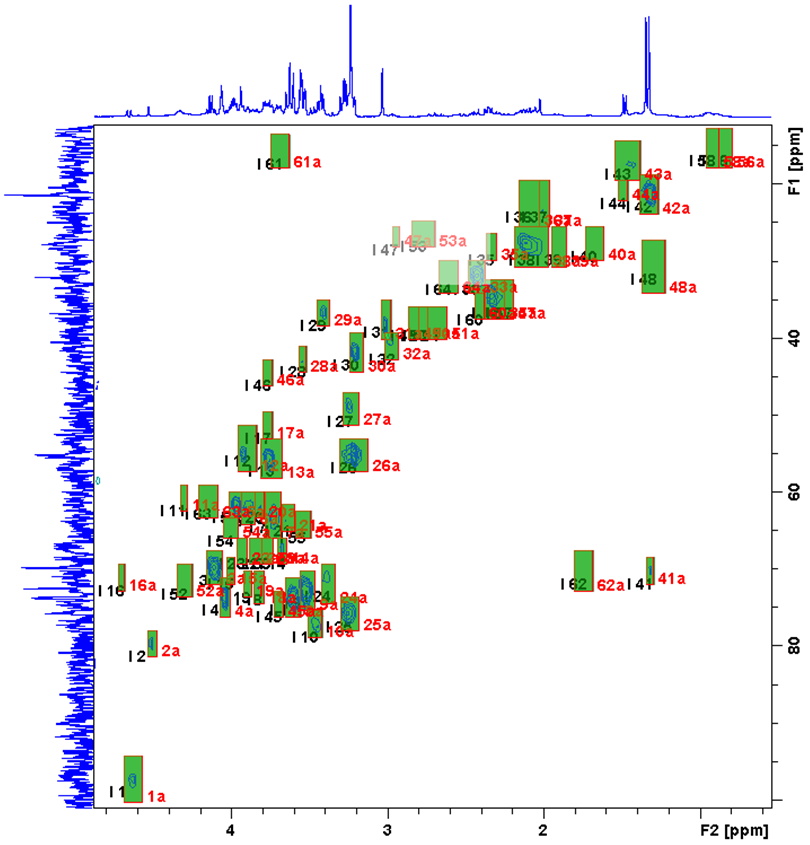
**

**B**

**
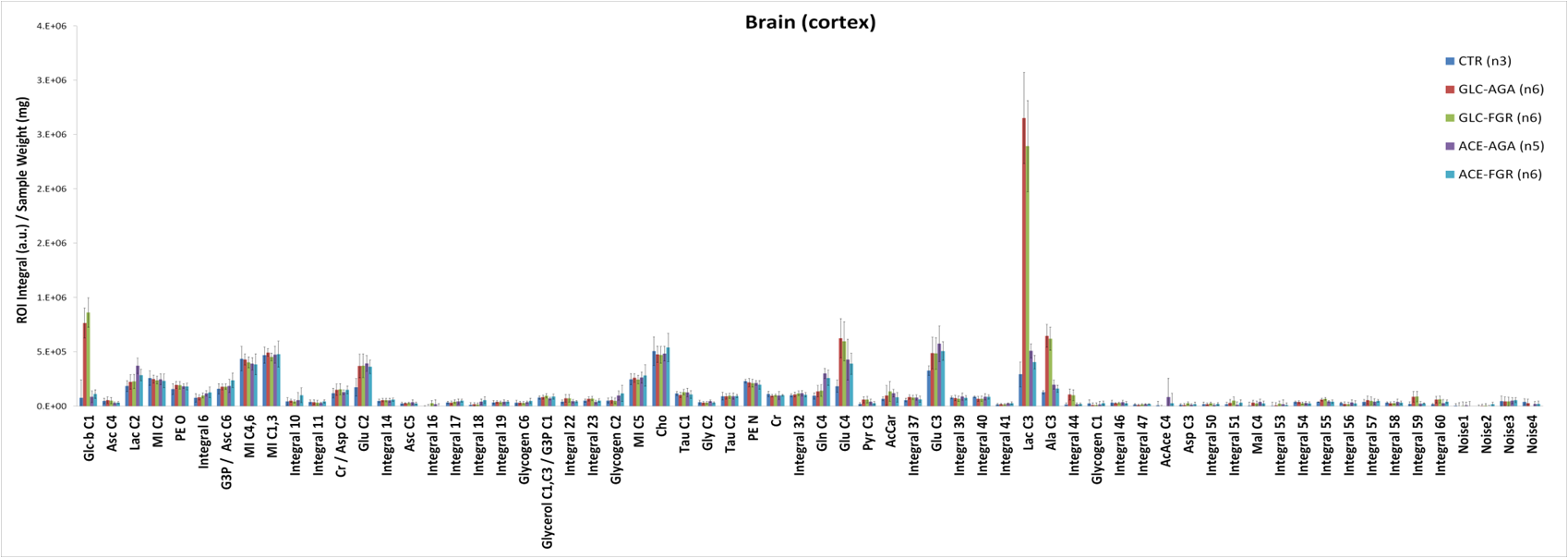
**

**C**

**
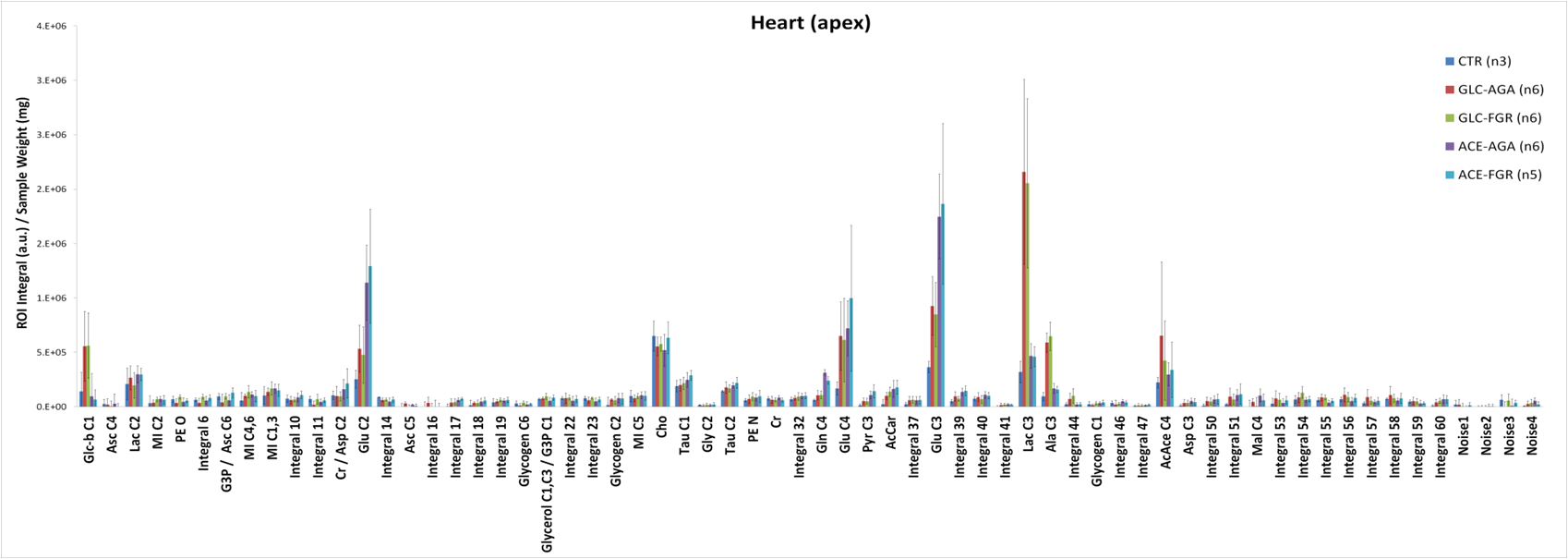
**

**S1 Fig. ROIs for quantification of 2D ^1^H-^13^C HSQC spectra**. A total of 60 metabolite peaks (and 4 noise regions) were included in the template (**A**), quantified by integration and normalized to sample weight for brain (**B**) and heart (**C**) tissues.
