# Supplementary material for: Assessment of prenatal cerebral and cardiac metabolic changes in a rabbit model of fetal growth restriction based on 13C-labelled substrate infusions and ex vivo multinuclear HRMAS
Source: PLoS One. 2018 Dec 27;13(12):e0208784. doi: 10.1371/journal.pone.0208784 (PMC6307735; doi:10.1371/journal.pone.0208784)
Supplement: S2 Fig — Values are displayed as normalized ROI integrals (average ±SD) for GLC and ACE groups, referenced to the CTR group (%). Significant differences between AGA and FGR subjects are indicated (* p<0.05, two-tailed unpaired t-Test). Lac C3’ (synthesized from pyruvate C3, essentially derived from 1-13C-glucose) = Lac C3 –Lac C2; Lac C2C3 (synthesized from 1:1 pool of pyruvate C2 and C3, essentially derived from 1:1 pool of malate C2 and C3 shuttled from the mitochondria) = 2 · Lac C2. (DOCX) [file pone.0208784.s009.docx]

**
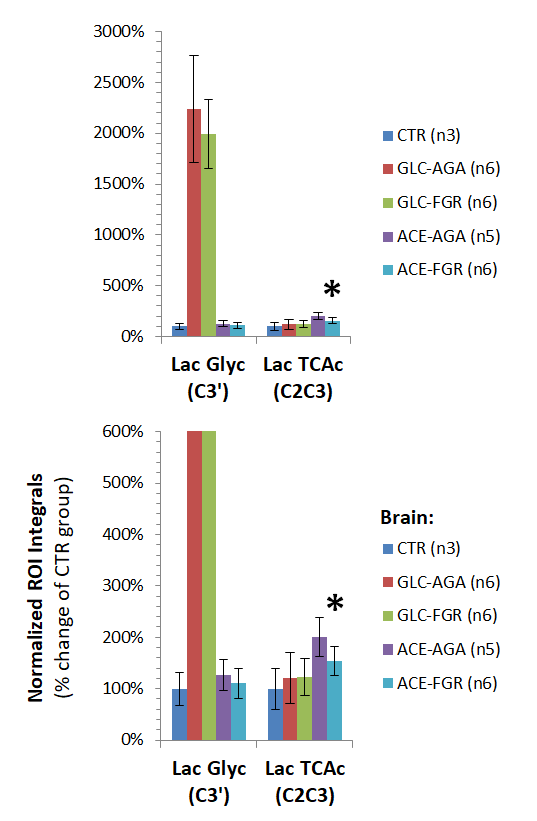
**

**S2 Fig. Estimations of brain ^13^C-lactate enrichments from glycolysis and TCAc**. Values are displayed as normalized ROI integrals (average ±SD) for GLC and ACE groups, referenced to the CTR group (%). Significant differences between AGA and FGR subjects are indicated (* p<0.05, two-tailed unpaired t-Test). Lac C3’ (synthesized from pyruvate C3, essentially derived from 1-^13^C-glucose) = Lac C3 – Lac C2; Lac C2C3 (synthesized from 1:1 pool of pyruvate C2 and C3, essentially derived from 1:1 pool of malate C2 and C3 shuttled from the mitochondria) = 2 · Lac C2.
