# Supplementary material for: Assessment of prenatal cerebral and cardiac metabolic changes in a rabbit model of fetal growth restriction based on 13C-labelled substrate infusions and ex vivo multinuclear HRMAS
Source: PLoS One. 2018 Dec 27;13(12):e0208784. doi: 10.1371/journal.pone.0208784 (PMC6307735; doi:10.1371/journal.pone.0208784)
Supplement: S3 Fig — Brain (A) and heart (B) tissues resampled from one of the AGA-ACE subjects used for the main experiments. Linear adjustments for Gln C4 (blue), Lac C2 (red) and Lac C3 (green) confirmed a slight accumulation of these metabolites over 5.2 h. (DOCX) [file pone.0208784.s010.docx]

**
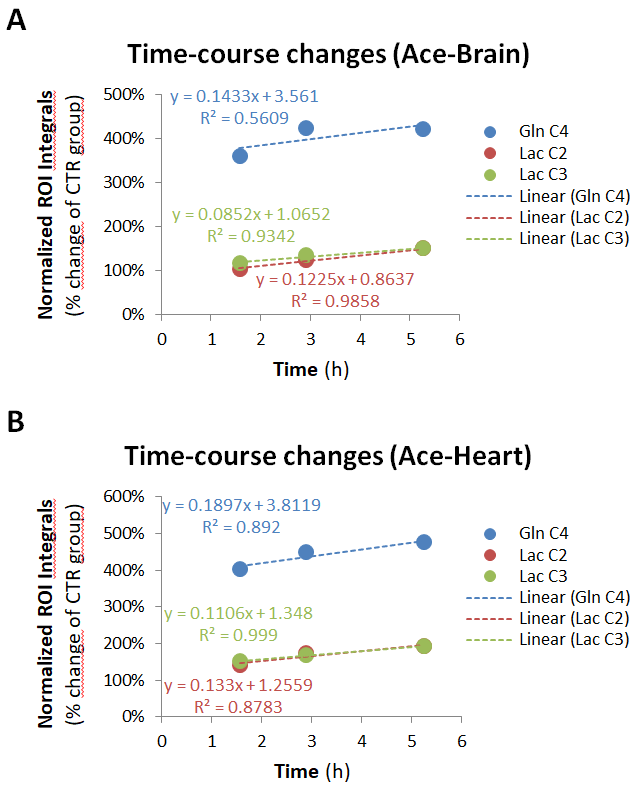
**

**S3 Fig. Time-course changes detectable in 2D ^1^H-^13^C spectra**. Brain (**A**) and heart (**B**) tissues resampled from one of the AGA-ACE subjects used for the main experiments. Linear adjustments for Gln C4 (blue), Lac C2 (red) and Lac C3 (green) confirmed a slight accumulation of these metabolites over 5.2 h.
