# Supplementary material for: Assessment of prenatal cerebral and cardiac metabolic changes in a rabbit model of fetal growth restriction based on 13C-labelled substrate infusions and ex vivo multinuclear HRMAS
Source: PLoS One. 2018 Dec 27;13(12):e0208784. doi: 10.1371/journal.pone.0208784 (PMC6307735; doi:10.1371/journal.pone.0208784)
Supplement: S4 Fig — Values based on the final 1H-CPMG spectrum acquired in each HRMAS session (A), indicating metabolite peak areas normalized to sample weight (average ±SD). Significant differences between FGR and AGA fetuses detected only for the estimated 13C-labelled lactate pool, 13CH3 (* p<0.05, two-tailed unpaired t-Test). Difference between the metabolite quantifications in A and the respective levels obtained from the initial 1H-CPMG sequence (Fig 4) (B). Sample sizes (n): heart glutamine, 6 AGA and 5 FGR; brain lactate, 5 AGA and 6 FGR. Lac 13CH3 = 2· ½ Lac 13CH3; Lac Total = Lac CH3 + Lac 13CH3. (DOCX) [file pone.0208784.s011.docx]

**
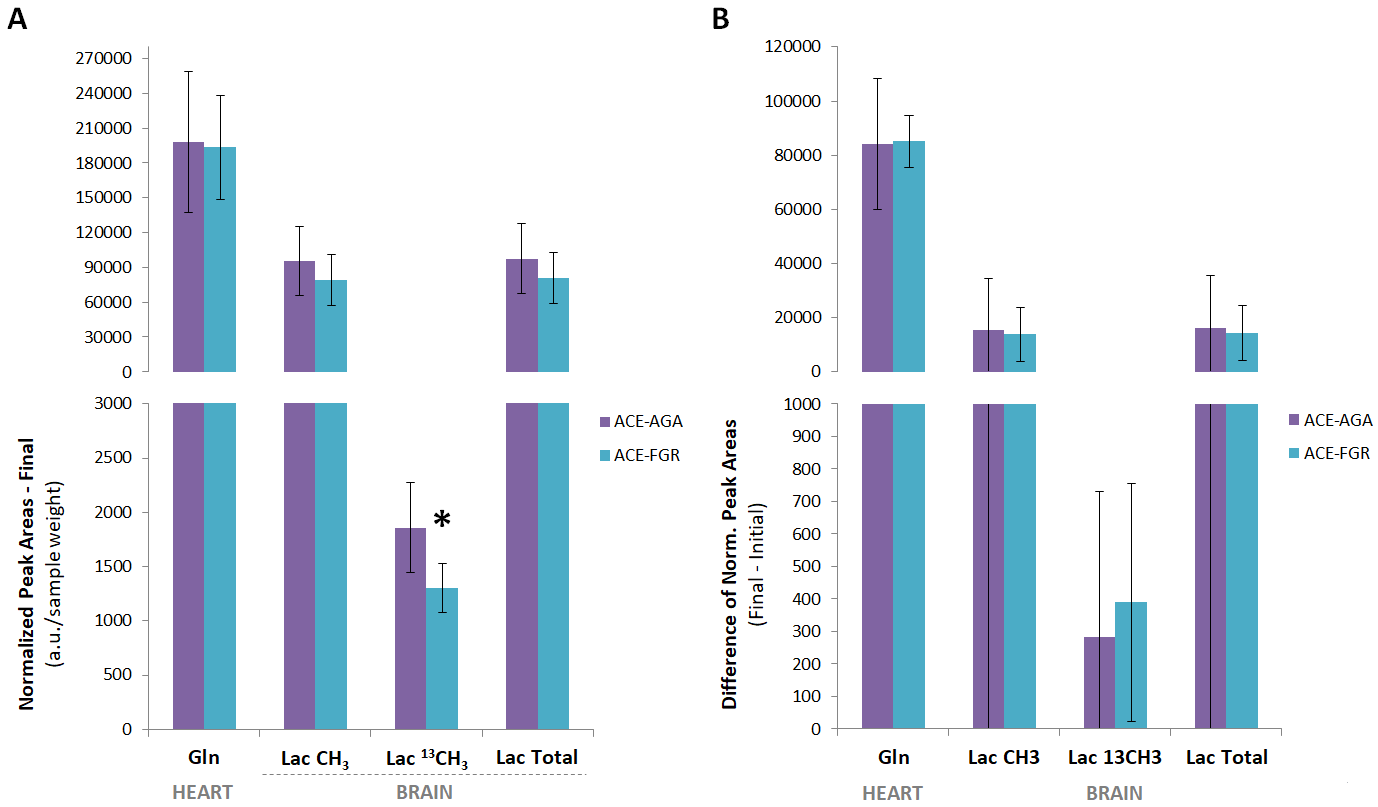
**

**S4 Fig. Quantification of heart glutamine and brain lactate pools based on the final ^1^H-CPMG spectra of the ACE group**. Values based on the final ^1^H-CPMG spectrum acquired in each HRMAS session (**A**), indicating metabolite peak areas normalized to sample weight (average ±SD). Significant differences between FGR and AGA fetuses detected only for the estimated ^13^C-labelled lactate pool, ^13^CH_3_ (* p<0.05, two-tailed unpaired t-Test)_._ Difference between the metabolite quantifications in A and the respective levels obtained from the initial ^1^H-CPMG sequence (Fig. 4) (**B**). Sample sizes (n): heart glutamine, 6 AGA and 5 FGR; brain lactate, 5 AGA and 6 FGR. Lac ^13^CH_3_ = 2· ½ Lac ^13^CH_3_ ; Lac Total = Lac CH_3_ + Lac ^13^CH_3_.
